# Sympathetic Innervation Induced in Engrafted Engineered Cardiomyocyte Sheets by Glial Cell Line Derived Neurotrophic Factor *In Vivo*

**DOI:** 10.1155/2013/532720

**Published:** 2013-08-28

**Authors:** Xian-ming Fu, Jong-Kook Lee, Keiko Miwa, Tatsuya Shimizu, Yoshiko Takagishi, Masumi Hirabayashi, Kazuhiko Watabe, Akihiko Usui, Itsuo Kodama, Yuichi Ueda

**Affiliations:** ^1^Department of Cardiac Surgery, Graduate School of Medicine, Nagoya University, 65 Tsurumai, Showa-ku, Nagoya, Aichi 466-8550, Japan; ^2^Department of Cardiovascular Regenerative Medicine, Osaka University Graduate School of Medicine, 2-2 Yamadaoka, Suita, Osaka 565-0871, Japan; ^3^Department of Medical Laboratory Science, Faculty of Health Sciences, Hokkaido University, Kita-12 Nishi-5, Kita-ku, Sapporo, Hokkaido 060-0812, Japan; ^4^Institute of Advanced Biomedical Engineering and Science, Tokyo Women's Medical University, 8-1 Kawada-cho, Shinjuku-ku, Tokyo 162-8666, Japan; ^5^Department of Genetics, Research Institute of Environmental Medicine, Nagoya University, Furo-cho, Chikusa-ku, Nagoya, Aichi 464-8601, Japan; ^6^Center for Genetic Analysis of Behavior, National Institute for Physiological Sciences, 5-1 Higashiyama, Myodaiji-cho, Okazaki, Aichi 444-8787, Japan; ^7^ALS and Neuropathy Project, Tokyo Metropolitan Institute of Medical Science, 2-1-6 Kamikitazawa, Setagaya-ku, Tokyo 156-8506, Japan; ^8^Nagoya University, Furo-cho, Chikusa-ku, Nagoya, Aichi, 464-8601, Japan

## Abstract

The aim of myocardial tissue engineering is to repair or regenerate damaged myocardium with engineered cardiac tissue. However, this strategy has been hampered by lack of functional integration of grafts with native myocardium. Autonomic innervation may be crucial for grafts to function properly with host myocardium. In this study, we explored the feasibility of *in vivo* induction of autonomic innervation to engineered myocardial tissue using genetic modulation by adenovirus encoding glial cell line derived neurotrophic factor (GDNF). GFP-transgene (control group) or GDNF overexpressing (GDNF group) engineered cardiomyocyte sheets were transplanted on cryoinjured hearts in rats. Nerve fibers in the grafts were examined by immunohistochemistry at 1, 2, and 4 weeks postoperatively. Growth associated protein-43 positive growing nerves and tyrosine hydroxylase positive sympathetic nerves were first detected in the grafts at 2 weeks postoperatively in control group and 1 week in GDNF group. The densities of growing nerve and sympathetic nerve in grafts were significantly increased in GDNF group. No choline acetyltransferase immunopositive parasympathetic nerves were observed in grafts. In conclusion, sympathetic innervation could be effectively induced into engrafted engineered cardiomyocyte sheets using GDNF.

## 1. Introduction

Recently, myocardial regeneration has been expected as a new therapeutic strategy for severe heart failure. To date, numerous studies have been reported demonstrating improvement of heart function in support of this goal [1, 2]. However, functional integration of graft with native myocardium is still an unsolved issue.

In order to conduct myocardial regeneration therapy successfully, appropriate integration with host myocardial tissue will be crucial. Transplanted engineered myocardial tissue without innervation may not function appropriately in accordance with host, since normal cardiac tissue is properly innervated, and its function is precisely regulated by the systemic autonomic nervous system [3]. Therefore, to achieve functional integration with host myocardium, autonomic innervation of transplanted myocardial engineered tissue should be important.

It is well known that neuronal function and innervation are regulated by target organ-derived neurotrophic factors [4]. Therefore, neurotrophic factors have been extensively investigated in animal models of nerve injury to further enhance and accelerate the process of nerve regeneration and functional recovery [5]. A member of the transforming growth factor superfamily, GDNF, has been shown to promote the survival and function of several neuronal populations in the peripheral nervous system [6, 7]. Furthermore, we demonstrated that GDNF effectively promoted the sympathetic neuron outgrowth to cocultured cardiomyocytes and played an important role in inducing cardiac sympathetic innervation [8, 9]. In the present study, we explored the feasibility of induction of autonomic innervation into transplanted engineered cardiomyocyte sheets by adenoviral overexpression of GDNF in the graft tissue.

## 2. Materials and Methods

All animal experiments were performed in accordance with the Guide for the Care and Use of Laboratory Animals published by the US National Institutes of Health (NIH Publication no. 85-23, revised 1996) and approved by the Animal Care and Use Committee of Nagoya University (Protocol no. 24061).

### 2.1. Isolation of Neonatal Rat Ventricular Cardiomyocyte and Construction of Engineered Cardiomyocyte Sheets

Primary cultures of neonatal cardiomyocytes were prepared as reported previously [10]. Briefly, ventricles from 1- to 3-day-old GFP-positive Wistar neonatal rats were digested at 37°C in Hank's balanced salt solution containing collagenase (Worthington Biochemical Corporation, Lakewood, NJ, USA). Isolated cells were suspended in culture medium M199 (Gibco BRL, Carlsbad, CA, USA) containing 10% fetal bovine serum, 0.2% penicillin-streptomycin, and 2.7 mmol/L glucose. Cells were seeded at a cell density of 3.0 × 10^5^/cm^2^ onto temperature-responsive culture dishes (CellSeed, Tokyo, Japan) and incubated at 37°C in a humidified atmosphere with 5% CO_2_. On the next day, 2 *μ*L phosphate buffered saline (PBS) containing adenovirus encoding GDNF (AdGDNF) (5 to 15 m.o.i) or PBS only (used as control) was added to the medium of culture dishes, respectively, and cardiomyocytes were continuously cultured for another 3 days. To release confluent cells as a cell sheet from the bottom of culture dishes, cells were incubated at 20°C. Engineered cardiomyocyte sheets were detached spontaneously within 1 hour and floated into the aqueous media. Immediately after detachment, the cell sheets were gently aspirated into the tip of a 10 mL pipet and transferred onto appropriate culture surfaces. Once placed, the medium was dropped onto the center of the sheet to spread folded parts of the transferred engineered cardiomyocyte sheets. After spreading cell sheets, the medium was then aspirated to adhere the cell sheet to the culture surface. To layer cell sheets, another cardiomyocyte sheet was transferred into the first dish in the same way. The second sheet was positioned just above the first sheet. Identical procedures were repeated to layer the third sheets.

### 2.2. Animal Model and Engineered Cardiomyocyte Sheets Transplantation

Male Wistar rats (8–10 weeks old, weight 222–325 g) were used to create heart cryoinjury model. Rats were anaesthetized with intraperitoneal injection of pentobarbital sodium (50 mg/kg), then intubated and mechanically ventilated with room air. Under aseptic condition, thoracotomy was performed through the left fifth intercostal space, and the heart was exposed. To create heart cryoinjury, a steel cryoprobe (3.5 mm in diameter), soaked in liquid nitrogen, was applied to the beating heart in the region of the anterior wall of the left ventricle for 10 seconds. Then the cryoprobe was removed, and the frozen tissue was allowed to thaw for 10 minutes. The freeze-thaw procedure was repeated twice. Triple-layered engineered cardiomyocyte sheets were then transplanted onto the injured anterior wall of the left ventricles. After 15 minutes, air was evacuated from the cavity, and the chest was closed, and then spontaneous normal respiration was restored. The rats were maintained under postoperative care and were given tacrolimus (Astellas Pharma Inc, Tokyo, Japan) at 10 mg/kg/d on the day before surgery transplantation, then on every day after surgery. They were euthanized at three time points (1, 2, and 4 weeks postoperatively).

### 2.3. Histology Examination

Engineered cardiomyocyte sheets, harvested 4 days after culture, were processed for GDNF immunostaining to confirm adenoviral transfection. Rat hearts were obtained at 1, 2, and 4 weeks postoperatively and rapidly placed in PBS containing paraformaldehyde (4%, adjusted to pH 7.4) at 4°C for 2 hours, washed in PBS, and sequentially transferred to graded (10, 20, and 30%) solutions of sucrose in PBS for 4 hours in each concentration. Hearts were embedded in Tissue-Tek-II OCT compound (Sakura Finetek Japan, Tokyo, Japan) and frozen on dry ice and then were cryosectioned and stained with primary antibodies for *α*-actinin (AA) (rabbit monoclonal, Sigma-Aldrich), growth associated protein-43 (GAP43, mouse monoclonal, Sigma-Aldrich), tyrosine hydroxylase (TH, mouse monoclonal, Sigma-Aldrich), neurofilament-M (NFM-M, rabbit polyclonal, Chemicon International), choline acetyltransferase (ChAT, mouse monoclonal, Sigma-Aldrich), and GDNF (goat polyclonal, R&D system, Minneapolis, MN). The sections were incubated with secondary antibodies conjugated with Alexa 568, 633 (Molecular Probes, Carlsbad, CA, USA). All confocal microscopic images were obtained using LSM 510 microscope (Carl Zeiss, Jena, Germany). The densities of GDNF or AA positive cardiomyocytes, GAP43 or TH positive nerve fibers were evaluated using Image-Pro Plus software (Media Cybernetics, Inc., Bethesda, MD, USA).

### 2.4. Quantitative Analysis of Sympathetic Innervation

We measured innervation in cardiac grafts at 3 time points, in 6 sections per animal (*n* = 5 animals per data). In each section, the six fields that contained the most nerve fibers were analyzed. We defined that the nerve density was the ratio between the total area of nerves and the total engrafted cardiomyocytes area by ImageJ software, as described previously [11].

### 2.5. Statistical Analysis

Data analyses were performed with SPSS for Windows (version 16.0). All data were described as mean ± standard deviation (SD). Comparison between two groups was analyzed using Student's *t*-test. A value of *P* < 0.05 was considered as statistically significant.

## 3. Results

### 3.1. GDNF Overexpression *In Vitro* and *In Vivo*

**Figure 1:**
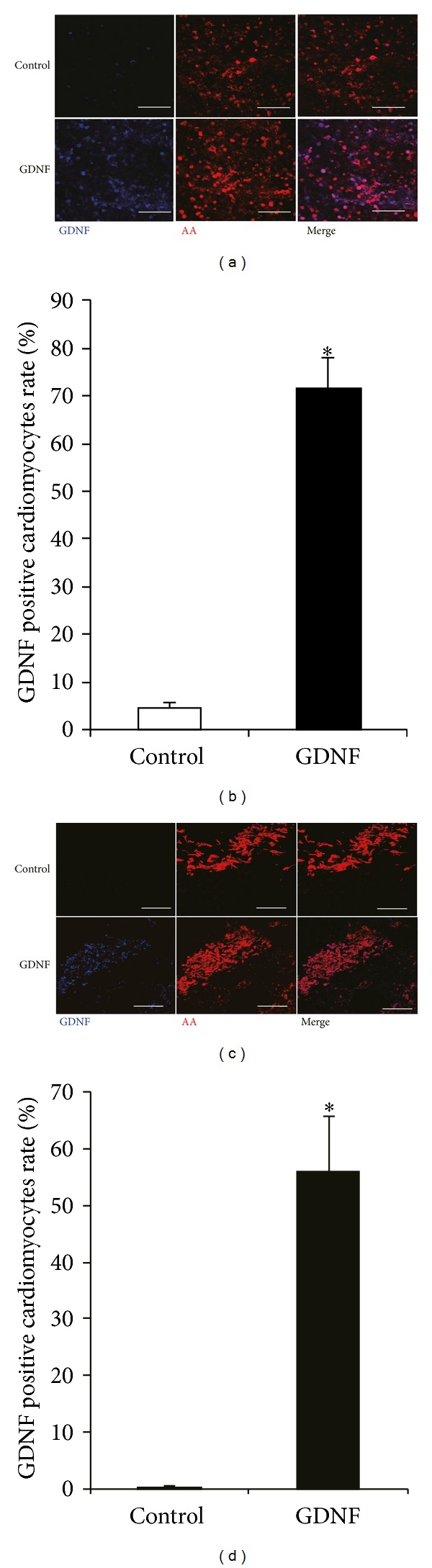
Overexpression of GDNF *in vitro* and *in vivo*. (a) Representative images of immunofluorescent staining for *α*-actinin (AA) and glial cell line derived neurotrophic factor (GDNF) in the control and GDNF cardiomyocytes after culture for 4 days. (b) Quantitative analysis of GDNF positive cells area in the control and GDNF cardiomyocytes (*P < 0.05, versus control, *n* = 4). (c) Representative images of immunofluorescent staining for *α*-actinin (AA) and GDNF in the control and GDNF grafts at 4 weeks after transplantation. (d) Quantitative analysis of GDNF positive cells area in the control and GDNF grafts (*P < 0.05, versus control, *n* = 4). Scale bars indicate 100 *μ*m. AA: *α*-actinin; GDNF: glial cell line derived neurotrophic factor.

To confirm adenoviral transfection and gene expression, AdGDNF transfected cardiomyocytes or control cardiomyocytes were harvested to perform immunofluorescence staining after 4 days of culture. GDNF protein was abundantly expressed in GDNF group cardiomyocytes, while GDNF was faintly detected in the control cardiomyocytes (Figure 1(a)). The ratio of GDNF-positive cells area was 4.6 ± 1.2% in control group and 71.7 ± 6.3% in GDNF group (Figure 1(b)) (*P* < 0.05, versus control, *n* = 4). In addition, to examine the long-term GDNF overexpression by adenoviral gene transfer, we also conducted immunostaining for cardiomyocyte sheets 4 weeks after transplantation. Abundant GDNF protein was observed in GDNF grafts, while no obvious GDNF was detected in control grafts (Figure 1(c)). Quantitative analyses of the relative GDNF positive cells area in control and GDNF grafts were 0.1 ± 0.2% and 56.0 ± 9.8% (Figure 1(d)) (*P* < 0.05, versus control, *n* = 4). These results suggested that stable GDNF overexpression of cardiomyocytes could be achieved *in vitro* and *in vivo* by genetically modifying cardiomyocytes.

### 3.2. Morphology of Cardiac Cryoinjury Model and Engrafted Engineered Cardiomyocyte Sheets

**Figure 2:**
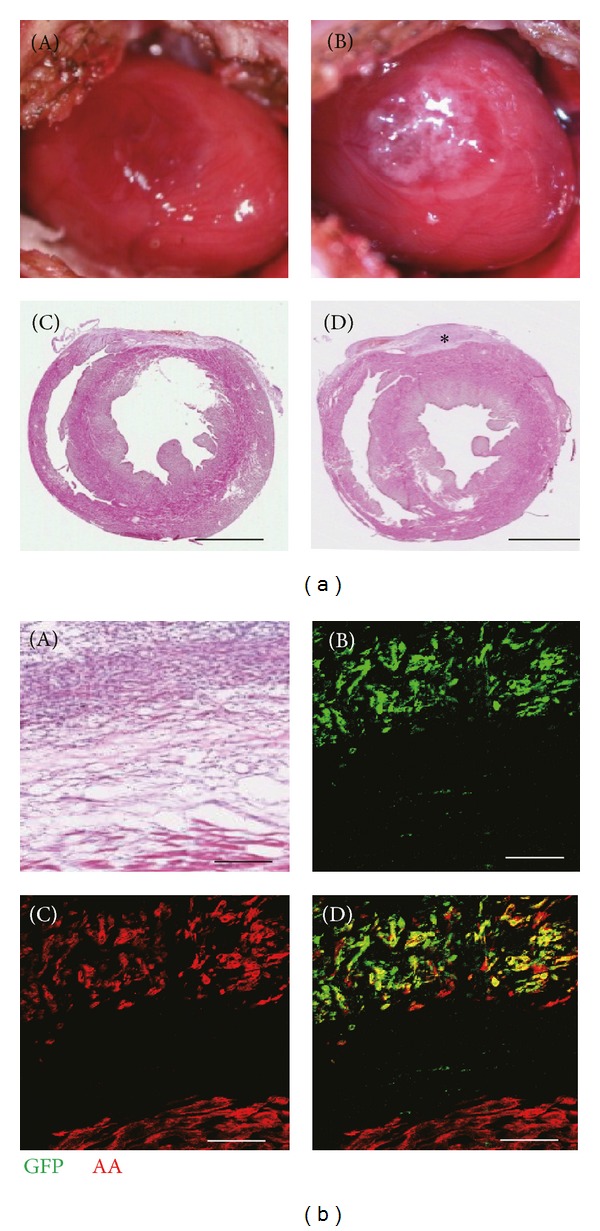
Morphology of cardiac cryoinjury model and engrafted engineered cardiomyocyte sheets. (a): (A) and (B) show cryoinjury on the epicardial surface of the left ventricle and transplantation with engineered cardiomyocyte sheets. (C) and (D) show representative hematoxylin/eosin staining of cross section of cryoinjured hearts without or with cardiomyocyte sheets transplantation 4 weeks after operation. Asterisk shows cardiomyocyte sheets transplanted on the epicardial surface of cryoinjured heart. (b): (A) shows representative hematoxylin/eosin staining of cardiomyocyte sheets 4 weeks after transplantation; (B)–(D) show the serial sections immunolabeling with *α*-actinin (AA, red),which marks cardiomyocytes, and GFP (green), which marks grafts. The grafted cardiomyocyte sheets can be identified by double positive for AA and GFP. Scale bars: 2 mm in Figures 2(a)C and 2(a)D; 200 *μ*m in Figure 2(b). AA: *α*-actinin.

In order to avoid variation of size, depth, and location of myocardial injury, we used a cardiac cryoinjury model as previously reported [12]. In the cryoinjured hearts, a round myocardial injury was observed on the epicardial surface of the left ventricle and covered with cardiomyocyte sheets (Figures 2(a)(A) and 2(a)(B)). Fibrotic tissues with 0.21 to 0.36 mm depth were observed on the epicardial surface at 4 weeks after operation (Figure 2(a)(C)). On the other hand, the transplanted engineered cardiomyocyte sheets were observed on the surface of cryoinjured myocardium and surrounded with fibrotic tissue (Figure 2(a)(D)). In order to track the transplanted cardiomyocyte sheets, GFP-transgenic rat neonatal ventricular cardiomyocytes were used. Four weeks after operation, engrafted cardiomyocyte sheets could be detected on the surface of cryoinjured heart by immunofluorescence staining, which show double positive for *α*-actinin and GFP (Figure 2(b)). These results indicated that the procedure used in the present study could create heart cryoinjury and transplanted cardiomyocytes could survive 4 weeks after transplantation.

### 3.3. Neural Growth in Engrafted Engineered Cardiomyocyte Sheets

**Figure 3:**
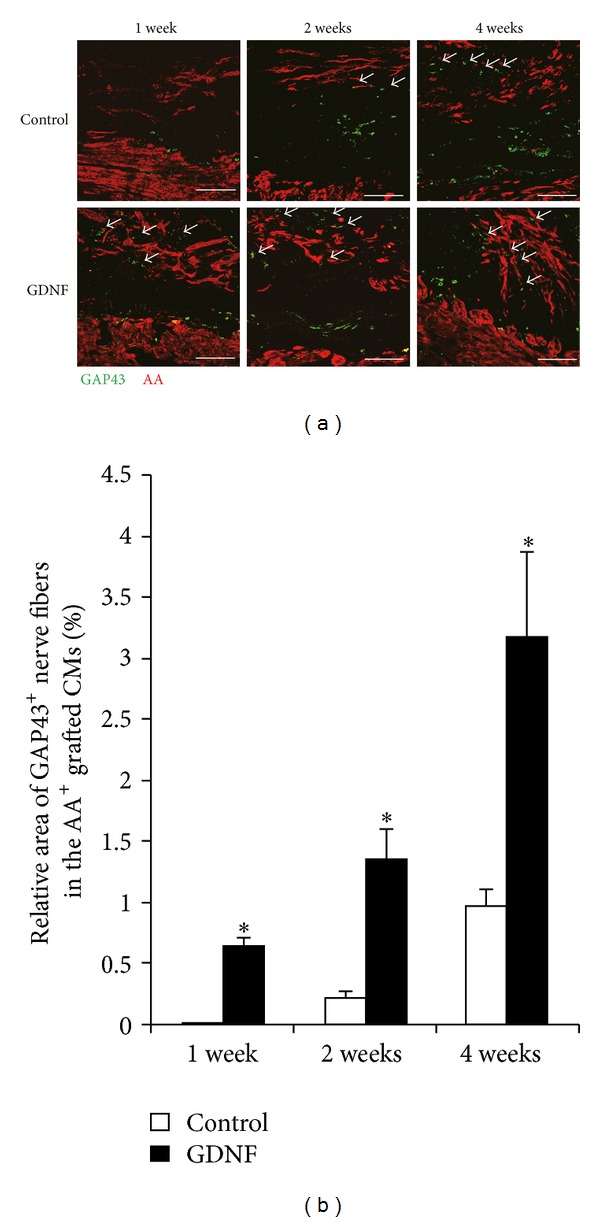
Neural growth in engrafted engineered cardiomyocyte sheets. (a) Representative images of immunofluorescent staining for *α*-actinin (AA) and growth associated protein 43 (GAP43) in the control and GDNF grafts at 1, 2, and 4 weeks after cardiomyocyte sheets transplantation. Arrows indicate growing nerves in the grafts. (b) Quantitative analysis of GAP43 positive nerve area in the control and GDNF cardiomyocyte sheet grafts (*P < 0.05, versus control, *n* = 5). Scale bars indicate 200 *μ*m. AA: *α*-actinin; GAP43: growth associated protein 43.

To examine the autonomic innervation in engrafted engineered cardiomyocyte sheets, we first investigated the growing nerves in the grafts at 1, 2, and 4 weeks after operation by immunostaining for GAP43, a maker for neural growth. At peri-injured areas, abundant GAP43 immunopositive nerves were detected in both group grafts at three time points. No GAP43 positive nerve fibers were observed in the control grafts until 2 weeks after transplantation, but some were observed 1 week in GDNF grafts (Figure 3(a)). GAP43 positive nerves were increased in the both group grafts over time. However, much more nerves were observed in GDNF group compared with control group at the same time point. The observation was confirmed by the measurement of the density of growing nerve fibers in the grafted cardiomyocyte sheets (Figure 3(b)) (*P* < 0.05, versus control, *n* = 5). These results indicated that GDNF effectively promoted neural growth in the grafts.

### 3.4. Sympathetic Innervation in Engrafted Engineered Cardiomyocyte Sheets

**Figure 4:**
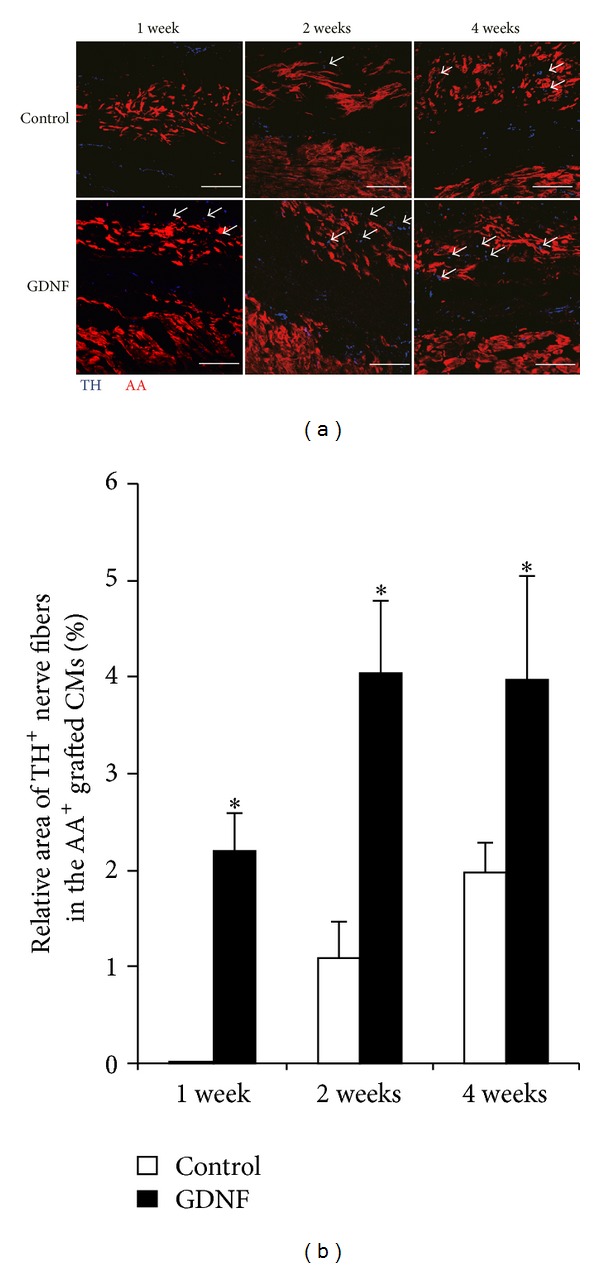
Sympathetic innervation in engrafted engineered cardiomyocyte sheets. (a) Representative images of immunofluorescent staining for *α*-actinin (AA) and tyrosine hydroxylase (TH) in the control and GDNF grafts at 1, 2, and 4 weeks after cardiomyocyte sheets transplantation. Arrows indicate sympathetic nerves in the grafts. (b) Quantitative analysis of TH positive nerve area in the control and GDNF cardiomyocyte sheet grafts (*P < 0.05, versus control, *n* = 5). Scale bars indicate 200 *μ*m. AA: *α*-actinin; TH: tyrosine hydroxylase.

To further investigate the type of nerve fibers in the engrafted engineered cardiomyocyte sheets, we did immunostaining for TH and ChAT to identify sympathetic and parasympathetic nerve in the grafts at 1, 2, and 4 weeks postoperatively. TH positive sympathetic nerve fibers were only subtly observed in the grafts until 2 weeks after transplantation in the control group, while the nerve fibers were clearly detected as early as 1 week after transplantation in GDNF group. More nerve fibers were observed over time in both group grafts (Figure 4(a)). Quantitative analysis of TH positive nerves area in the grafts shows that much more sympathetic nerves were observed in the GDNF group grafts compared with the control at the same time points (Figure 4(b)) (*P* < 0.05, versus control, *n* = 5).

**Figure 5:**
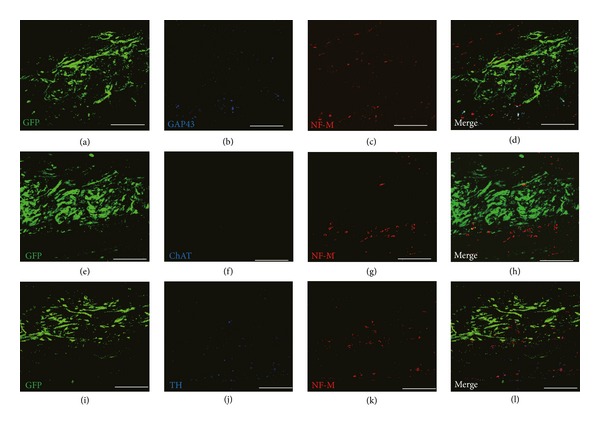
Patterns of growing nerves (GAP43), sympathetic nerves (TH), parasympathetic nerves (ChAT), and autonomic nerves (NF-M) in the grafts. (a)–(d), (e)–(h), and (i)–(l) show representative images of triple immunostaining for NF-M/GAP43/GFP, NF-M/ChAT/GFP, and NF-M/TH/GFP in the GDNF grafts at 4 weeks after transplantation. Scale bar indicates 200 *μ*m. GAP43: growth associated protein 43; TH: tyrosine hydroxylase; ChAT: choline acetyltransferase; NF-M: neurofilament-M.

On the other hand, no ChAT immunoreactive parasympathetic nerves were observed either at peri-infarct area or in the engrafted cardiomyocyte sheets (samples were obtained from GDNF cardiomyocyte sheets transplanted rats), while the pattern of GAP43 positive nerves and TH positive nerves was similar with that of NF-M positive nerves in the same sample (Figure 5). These results were confirmed in four independent experiments. These findings indicated that sympathetic, but not parasympathetic, innervation in engrafted engineered cardiomyocyte could be efficiently enhanced by GDNF.

## 4. Discussion

The present study showed a new technology to induce sympathetic innervation in engrafted engineered cardiomyocyte sheets. With the use of *in vitro* gene transfer strategy, overexpression of GDNF, the sympathetic innervation in grafts was significantly improved.

Recently, myocardial regeneration therapy is considered as a promising treatment for the patients with heart failure. To conduct cell therapy more safely, scaffold-free cardiac cell sheets techniques are engineered using temperature-responsive culture dishes. With this procedure, cells can be harvested as intact sheets, and the three-dimension tissues are constructed by layering these cell sheets [13]. Transplantation of cell sheets onto damaged hearts improved heart function in several animal models [14, 15]. However, long-term survival and functional integration of grafts with native myocardium are still two crucial issues for clinical application in future. Vascularization of graft is widely investigated and has been shown benefit for survival [16, 17]; however, little information is available for autonomic innervation in engrafted engineered myocardial tissue. In the present study, we used cell sheet technique to construct engineered triple-layered cardiomyocyte sheets and transplanted them onto the cryoinjured hearts. We demonstrated that sympathetic but not parasympathetic innervation in the transplanted engineered cardiomyocyte sheets at 2 weeks after transplantation and provided evidence that this process could be significantly promoted by genetically modifying cardiomyocytes to overexpress GDNF.

It is a promising strategy to combine gene therapy and tissue engineering or cell therapy for treatment of diseases. Previously, vascular growth has been successfully induced into tissue-engineered scaffolds by combination of VEGF overexpressing adipose-derived stromal cells and endothelial cells transplantation [18, 19]. In addition, transplantation of GDNF overexpressing Schwann cells has been reported to enhance regeneration of bilaterally transected erectile nerves in rats [20]. By similar strategy, in the present study, overexpression of GDNF in the graft tissue by adenovirus transfection effectively induced sympathetic innervation into the transplanted engineered myocardiocyte sheets.

GDNF has been shown to promote the survival and function of several neuronal populations in the peripheral nervous system [6, 7]. Recent findings have shown that GDNF was expressed in the hearts of murine embryos and neonates by quantitative RT-PCR [21] and upregulated after chemical sympathectomy in rats, suggesting a role in sympathetic nerve regeneration [22]. In addition, artemin, a neurotrophic factor of GDNF family, was shown to express along blood vessels in the early developmental stages and promote the development of sympathetic innervation of blood vessels [23, 24]. Previously, we have reported that GDNF enhanced sympathetic axon growth toward cardiomyocytes [8]. In this study, our results showed that GDNF also effectively promoted sympathetic innervation into transplanted engineered cardiomyocyte sheets *in vivo*.

It should be noted that the type of nerve innervating transplanted cardiomyocytes seems to be dependent upon the transplant site, as they are derived from the surrounding tissues. In the rat heart, the sympathetic nerves are distributed in the subepicardial layer throughout most surfaces and penetrate into myocardium along coronary arterial pathways, while parasympathetic nerves are mainly located around conducting system. In this study, engrafted engineered cardiomyocytes were innervated by TH positive sympathetic fibers but not parasympathetic nerves. Similar findings have been reported in previous studies about transplanted islets [25].

There are several limitations to the present study. First, it is worth to note that sympathetic hyperinnervation may cause life-threatening arrhythmias. In this study, we used GDNF to increase sympathetic innervation in transplanted cardiomyocyte sheets, and it may induce arrhythmias. To assess the potential risk of sympathetic innervation in engrafted cardiomyocyte sheets, we performed *in vivo* programmed electric stimulation at 4 weeks after transplantation; however, no induction of arrhythmia was detected (data not shown). Maybe our model would not be suitable for discussing arrhythmogenesis because the size of the cryoinjury is apparently too small to induce arrhythmias. Further examination is needed.

Second, in this study, we showed that sympathetic innervation occurs in engrafted cardiomyocytes and demonstrated this could be promoted by GDNF, although we did not present the evidence showing that sympathetic innervation can promote engrafted cardiomyocytes functionally integrated with host myocardium. Further functional assessment is needed.

In conclusion, our work has demonstrated that sympathetic innervation could be effectively induced into engrafted engineered cardiomyocyte sheets by GDNF. This study may be an important step to engineer functional myocardium in myocardial regeneration therapy.
